# Habitat loss for black flying foxes and implications for Hendra virus

**DOI:** 10.1007/s10980-023-01642-w

**Published:** 2023-04-05

**Authors:** Kelsee Baranowski, Nita Bharti

**Affiliations:** Department of Biology, Center for Infectious Disease Dynamics, The Pennsylvania State University, University Park, PA USA

**Keywords:** *Pteropus*, Hendra virus, Habitat loss, Deforestation

## Abstract

**Context:**

Environmental change impacts natural ecosystems and wildlife populations. In Australia, native forests have been heavily cleared and the local emergence of Hendra virus (HeV) has been linked to land-use change, winter habitat loss, and changing bat behavior.

**Objectives:**

We quantified changes in landscape factors for black flying foxes (*Pteropus alecto*), a reservoir host of HeV, in sub-tropical Queensland, Australia from 2000–2020. We hypothesized that native winter habitat loss and native remnant forest loss were greatest in areas with the most human population growth.

**Methods:**

We measured the spatiotemporal change in human population size and native ‘remnant’ woody vegetation extent. We assessed changes in the observed *P. alecto* population and native winter habitats in bioregions where *P. alecto* are observed roosting in winter. We assessed changes in the amount of remnant vegetation across bioregions and within 50 km foraging buffers around roosts.

**Results:**

Human populations in these bioregions grew by 1.18 M people, mostly within 50 km foraging areas around roosts. Remnant forest extent decreased overall, but regrowth was observed when policy restricted vegetation clearing. Winter habitats were continuously lost across all spatial scales. Observed roost counts of *P. alecto* declined.

**Conclusion:**

Native remnant forest loss and winter habitat loss were not directly linked to spatial human population growth. Rather, most remnant vegetation was cleared for indirect human use. We observed forest loss and regrowth in response to state land clearing policies. Expanded flying fox population surveys will help better understand how land-use change has impacted *P. alecto* distribution and Hendra virus spillover.

**Supplementary Information:**

The online version contains supplementary material available at 10.1007/s10980-023-01642-w.

## Introduction

Anthropogenic land-use change, driven by urban development, agricultural intensification, and resource needs, has led to the deforestation and degradation of ecosystems globally (Foley et al. 2005; Shellberg et al. 2016). Over 2.3 million square kilometers (230 M hectares (ha)) of global forest were lost between 2000–2012, mostly in the tropics, while only 0.8 million square kilometers (80 M ha) were gained through plantations or regrowth (Hansen et al. 2013). This extreme change has modified natural nutrient cycles, hydrological cycles, and regional climates (Postel et al. 1996; Kalnay and Cai 2003; Foley et al. 2005; Deo et al. 2009; Haddad et al. 2015). Repercussions of these changes have led to global increases in droughts, large wildfires, and floods at unprecedented scales (Westerling et al. 2006; Min et al. 2011; Baranowski et al. 2021; Department of Agriculture Water and the Environment 2021).

Human-driven land conversion has also removed or modified important habitats and resources for wildlife species. This often fragments native habitats (Tulloch et al. 2016; Shackelford et al. 2018) and can introduce exotic species, which create challenges for native species’ survival (Brook et al. 2003; Woinarski et al. 2015). Singapore has lost 95% of its primary forests since 1819 (Corlett et al. 1992) causing substantial extinction of forest species, particularly forest specialists (Brook et al. 2003). Similarly, Borneo has been a hot spot for land cover change, with intense conversion to oil palm plantations and logging causing a loss of at least 30% of native forest since 1973 (Gaveau et al. 2014). As a result, the nation has experienced significant declines in species richness, diversity, and animal abundance (Edwards et al. 2014). Other impacts on wildlife include range contraction (Shackelford et al. 2018), changing species compositions (Crooks 2002), changing species’ ecology and trophic interactions (Hebblewhite et al. 2005), and disease emergence (Patz et al. 2004; Gottdenker et al. 2014; Macdonald and Mordecai 2020).

As humans have invaded the natural world, the frequency of human-wildlife interactions has increased (Patz et al. 2004; Soulsbury and White 2015). These novel interactions carry the threat of zoonotic disease emergence, or emergence of pathogens that can cross-species barriers, representing a serious risk to global public health for humans and animal populations in close contact with humans. Many zoonotic viruses that cause severe diseases in humans, such as Ebola virus, Marburg virus, Hendra virus, Nipah virus, and SARS-CoV-1, have been the consequence of spillover events from bats (Plowright et al. 2015; Ruiz-Aravena et al. 2021). SARS-CoV-2, which likely spilled over from bats, caused over 6.7 million human deaths in its first three years post-emergence and had immense impacts on populations globally (Ruiz-Aravena et al. 2021; World Health Organization 2022). The World Health Organization (WHO) classified the diseases caused by several of these bat viruses as priority diseases for research (World Health Organization 2018), demonstrating an urgency to understand the mechanistic drivers of emergence for these and other zoonotic diseases.

Hendra virus is a henipavirus in the *Paramyxoviridae* family and is a priority disease designated by the WHO (Mahalingam et al. 2012; World Health Organization 2018). It was identified in 1994 in Queensland, Australia when twenty horses and one person died following a spillover event (Murray et al. 1995). Spillover likely occurs when a horse inhales or ingests the excreta of infected bats; all known Hendra virus spillovers have been the result of primary transmission from bat to horse (Plowright et al. 2011; Smith et al. 2014). Infected horses show respiratory and neurological symptoms and can transmit the virus to other horses, humans, and domestic animals (Murray et al. 1995; Plowright et al. 2011). Horses have a 75–80% natural case fatality rate (88 confirmed cases), although all suspected cases are euthanized, and humans have a 58% case fatality rate (4/7) (Queensland Government Business Queensland 2021). Another ecologically and genetically similar henipavirus, called Nipah virus, has caused hundreds of human cases with high mortality rates (range 40–70%) and is now endemic in southeast Asia (Epstein et al. 2020). Understanding the environmental drivers of Hendra virus spillover is a critical step towards characterizing the broader mechanisms of henipavirus spillover from bats.

Eby et al. 2023 characterized 25 years of environmental data to investigate the mechanisms of Hendra virus spillover and identify predictors of when 3 or more spillover events will occur in a year. Their findings suggest the clearing of key winter habitats, the consequence of human-induced land-use change, concurrent with the fissioning of flying fox populations to roost in agricultural areas led to clusters of spillovers after periods of food shortage. Becker et al. 2022 similarly showed pulses of Hendra virus shedding were most intense after food shortages in the previous spring and in areas where black flying foxes were newly overwintering. Flying foxes are typically nomadic, nocturnal nectarivores that commonly forage on small fruits, nectar, and pollen from members of the Proteaceae and Myrtaceae families, notably *Eucalyptus, Melaleuca, Banksia,* and *Corymbia* species (Eby 1991; Bell et al. 2021; Bradford et al. 2022). The climatic conditions that prompt eucalypt (tribe Eucalypteae within Myrtaceae) reproduction vary by location, soil type, and season (Law et al. 2000; Eby and Law 2008), creating a patchwork of resources across the landscape. Corresponding to this irregular flowering phenology, flying foxes historically migrated long distances across their range to track the seasonal flowering of eucalypts (Eby 1998; Roberts et al. 2012). This extreme mobility of flying foxes (Welbergen et al. 2020) makes them key pollinators and seed dispersers, important for the maintenance of forest ecosystem health (Marshall 1985; Eby 1998). However, seasonal variation in eucalypt blooming creates a resource bottleneck in winter months and consecutive winter food shortages were a key factor shown to drive Hendra virus spillover clusters after 2010 (Eby et al. 2023).

Australian flying fox roosting behavior has changed considerably in recent decades. In the mid-2000s, it was observed that small sub-populations of flying foxes were forgoing their historic migratory behavior and forming camps in urban or peri-urban areas (Van Der Ree et al. 2006; Plowright et al. 2011), particularly after periods of winter or spring food shortages (Eby et al. 2023). Recent studies on flying fox roost occupation show black and grey-headed flying foxes most commonly roost in urban and agricultural areas, with few roosts in protected areas (Timmiss et al. 2020; Eby et al. 2023). This change in bat behavior came after decades of extensive habitat loss (Eby et al. 2023) concurrent with an increase in the diversity and spatiotemporal availability of food in urban environments (Markus and Hall 2004; Meade et al. 2021; Yabsley et al. 2021), both stemming from human invasion and modification of bat habitats. Collectively, these anthropogenic forces are likely imposing a “push and pull” dynamic on flying fox populations and influencing roosting and foraging behavior (Yabsley et al. 2021). However, the impact of anthropogenic land pressure on black flying fox populations has been understudied given the species’ importance in pollinating eucalypt forests and role in Hendra virus spillover.

In bioregions where black flying fox populations were observed from 2012 to 2020, we quantify the changes in native remnant forest extent, native winter habitat extent, human population size, and black flying fox populations. We measure changes by bioregion, which are the primary levels of biodiversity classifications in Queensland (Queensland Government 2014), and within 50 km buffers around roosts, which is the nightly maximum foraging area (Eby 1991; New South Wales Department of Planning and Environment 2020). Given the role of human-induced environmental change in Hendra virus spillover (Eby et al. 2023), we hypothesize that winter habitat loss and remnant forest loss were largest in bioregions with the greatest amount of human population growth. Characterizing these spatiotemporal dynamics will inform our understanding of the impact of land-use change on black flying fox distribution and subsequent Hendra virus spillover in sub-tropical Queensland.

## Materials and methods

### Study area & black flying fox winter roosts

**Fig. 1:**
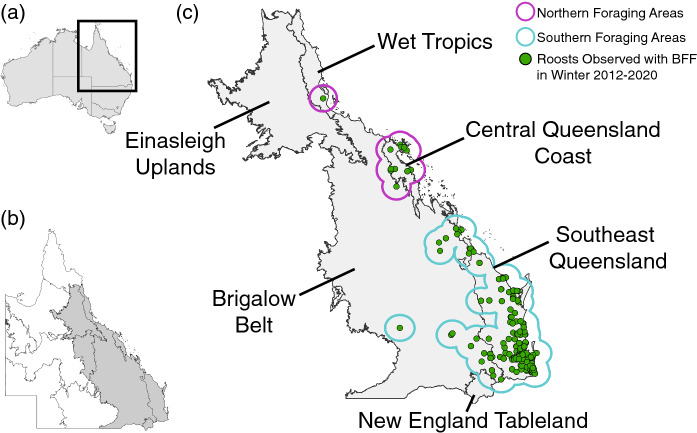
Study area. **a** Australia, denoting the state of Queensland. **b** All bioregions of Queensland with bioregions of interest shaded in grey. **c** Labeled bioregions of interest showing roosts occupied in winter by black flying foxes with green dots and 50 km buffers representing Northern and Southern Foraging areas denoted in purple and blue buffers, respectively

We focused on the state of Queensland, Australia (Fig. 1b) due to the presence of black flying foxes, high rates of historic deforestation (Bradshaw 2012; Evans 2016; Tulloch et al. 2016; Simmons et al. 2018) and the high incidence of Hendra virus spillover events (Mahalingam et al. 2012; Plowright et al. 2015). We retrieved surveys on flying fox roosts from Queensland’s National Flying Fox Monitoring Program (Australian Government of Agriculture, Water 2021). These surveys, completed by governments and volunteers, describe animal counts at nationally known or established roosts. They are focused on roosts used by grey-headed and spectacled flying foxes since these species are listed as ‘Vulnerable’ and ‘Endangered’ respectively. Although this focus biases the roost selection, black flying foxes and grey-headed flying foxes often co-roost where there is species sympatry (Welbergen et al. 2020) and counts of little red and black flying foxes are recorded when present. We included all roosts where black flying foxes were observed during any winter month (June, July, August) from 2012 to 2020 (Fig. 1c). We then buffered roost locations by 50 km to represent the maximum foraging areas for individuals (Fig. 1c). We also performed these analyses using buffer radii consistent with the average nightly foraging distance of 20 km (Roberts et al. 2012) and 80 km, the largest single night maximum flight observed by Yabsley et al. 2022, to investigate how loss varies across foraging radii (Figure S1; Table S3).

Buffers were spatially delineated into Northern and Southern foraging areas to account for variation in the flowering of diet species across this latitudinal gradient. For example, *Melaleuca quinquenervia* flowers in the southern foraging areas in winter (Eby and Law 2008), but not in the northern foraging areas. We analyzed roost occupancy trends after 2007, when annual surveys began, but only include counts of observed individuals after 2012, when the National Flying Fox Monitoring program implemented new methodology (Westcott et al. 2011; Australian Government of Agriculture, Water 2021). To limit the influence of sampling bias on observed counts, we use only the maximum number of black flying foxes counted in a single observation from each roost in each season. We refer to these as *roost counts*.

### Landscape changes

We used Worldpop Global Population datasets (WorldPop 2020) to assess the changes in human populations in these bioregions of interest. We acquired the 2000 and 2020 unconstrained, UN-adjusted 100-m grids of global human population counts. We masked these grids to the bioregions of interest and calculated the difference between the rasters to determine where populations increased, signaling human population growth, or decreased, signaling human population decline. We calculated the total increase in the number of people, the number of pixels that experienced human population growth within foraging areas of black flying fox occupied roosts, and the number of roosts within 100 m, or one pixel from where human population counts increased between 2000 and 2020.

We used the National Forest and Sparse Woody Vegetation Data Version 5 (Department of Environment and Science 2020) to assess the changes in woody vegetation and forest extent in the study area (Fig. 1). We merged rasters of forests and woody vegetation in Queensland by year and masked out our study area of interest. We assessed the primary loss of *remnant woody vegetation* or woody vegetation and forests inside areas denoted as ‘remnant’ from the Vegetation Management Regional Ecosystem (VMRE) map series (Queensland Herbarium 2019) in corresponding years. This method recapitulated remnant areas from the Remnant Vegetation of Queensland dataset, a map often used in studies of remnant deforestation in Queensland (Evans 2016; Simmons et al. 2018, 2021).

We obtained winter diet species lists from studies that observed black flying foxes winter feeding (Palmer 1997; Vardon et al. 2001; Markus and Hall 2004; Griffith 2020), the results of fecal analysis in winter months (Bell et al. 2021; Bradford et al. 2022), and known winter flowering diet species for grey-headed flying foxes in areas sympatric with black flying foxes (Eby and Law 2008; Eby et al. 2019). We compiled a major diet list and a possible diet list from these studies, to account for the species’ extensive geographic range and our uncertainty about their diet across Queensland. We restricted the major diet list to all flying fox winter diet species for which consumption was observed in the state of Queensland, and the possible diet list as all winter diet species for which consumption was observed in the states surrounding Queensland (Northern Territory or New South Wales). See supplemental material for list and descriptions of the major and possible winter diet species.

A previous study tested the capabilities of various satellite sensors to identify species of Queensland’s eucalypt vegetation remotely, but none were specific enough to distinguish between vegetation types (Baranowski et al. 2020). Instead, we use the Queensland Herbarium’s VMRE map series, provided by the Department of Environment and Science (Neldner et al. 2019; Queensland Herbarium 2019). These maps show Queensland’s remnant vegetation biennially from 1999 to 2019. They are created by integrating data from field surveys, aerial photography, satellite imagery, and other data including geology, soil mapping, and historical surveys (Neldner et al. 2019). Regional ecosystems are vegetation communities in a given bioregion that are consistently associated with a particular combination of geology, landform, and soil (Neldner et al. 2019). These maps can be associated with the Regional Ecosystem Description Database (Queensland Herbarium 2019) to provide highly detailed information about remnant vegetation across the landscape through time.

We define *winter habitats* as regional ecosystems dominated by at least one of the species included in the major diet or possible diet lists. (See the supplemental material for a detailed description of regional ecosystem selection.) We joined this list of regional ecosystems containing winter diet species (n = 3383) to each year of the VMRE maps and quantified the total hectares of these vegetation communities serially. Polygons were either homogeneous, with one regional ecosystem, or heterogeneous and contained up to five regional ecosystems. We included all polygons that had these regional ecosystems present in any proportion to account for all areas of potential winter habitat. We quantified the area of each winter habitat patch in hectares and scaled this area to account for the proportion of the patch that is composed of the regional ecosystem of interest. For example, if the regional ecosystem that contained the diet species was only 30% of that patch, we scaled the area of the polygon considered by 0.3. We summed the total area of the regional ecosystems of interest per patch and quantified the change in total hectares, number of patches, and mean patch size through time. We analyzed all spatial data in the Queensland Statewide Landcover and Trees Study (SLATS) Albers projection from the publicly available VMRE maps, processed using ArcGIS Pro 2.8 (ESRI 2020). Summary information was quantified and figures were visualized in R version 3.6 (R Core Team 2019).

We successively mapped cleared winter habitats between 1999 and 2019 to measure loss and fragmentation in these important patches. We used the Erase tool in ArcGIS Pro to identify areas that were present in year 1 and absent in year 2, signaling that area was cleared or degraded past the point of ‘remnant’ status by the Queensland Herbarium according to Neldner et al. 2019. We quantified the hectares lost between years and calculated the proportion of the patch lost as compared to the previous size. We classified patches as being “lost entirely” if the percent of the patch lost was 100% of the previous size.

## Results

### Landscape changes

**Fig. 2:**
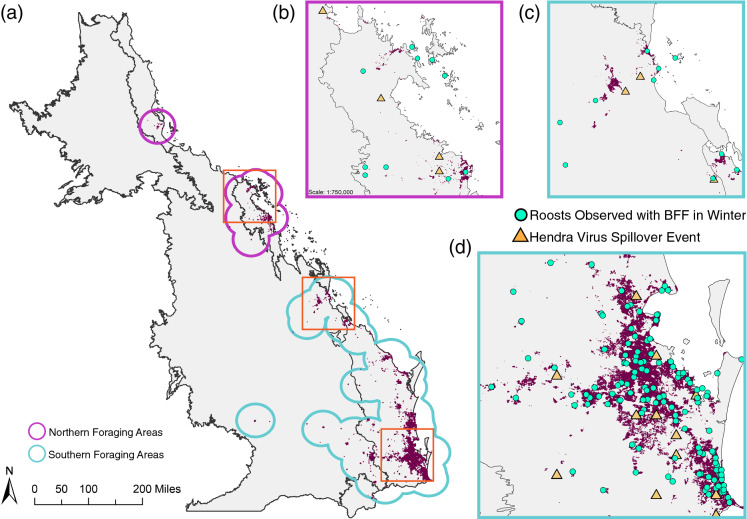
Areas of Human Population Growth 2000-2020 and Proximity to Roosts used by Black Flying foxes. **a** Bioregions of interest showing human population growth between 2000 and 2020 in dark purple pixels with Northern and Southern foraging areas outlined in purple and blue, respectively, and three red boxes identifying areas important for Hendra virus spillover are shown in panels (**b**-**d**). **b** Close up of Mackay, QLD, **c** close up of Rockhampton, QLD, **d** close up of Brisbane, QLD. For b-d, areas of human population growth are shown in dark purple, roosts occupied by black flying foxes during winter in green dots, and HeV spillovers in yellow triangles

Human populations have increased primarily along the coastline, with the Brisbane area of Southeast Queensland experiencing the most growth from 2000 to 2020 (Fig. 2a). Across all bioregions of interest, the human population increased by 1.18 million people (Table 1). Importantly, 86.4% (136,764/158,317 pixels) of that growth has been inside foraging areas around roosts occupied by black flying foxes in winter. Further, all 217 roosts considered here were within 100 m of a pixel where human population counts increased.

**Table 1:** Human population change by bioregion and roosts experiencing human population growth in foraging areas from 2012–2020

Bioregion	Brigalow Belt	Central Queensland Coast	Einasleigh Uplands	New England Tableland	Southeast Queensland	Wet Tropics
Net Change in Human Population Percent of Net Change in Human Population	+ 157,210 13%	+ 34,203 3%	+ 4,951 0%	+ 6,766 1%	+ 923,788 78%	+ 66,001 6%
Percent of Roosts with Human Growth in Foraging Areas (#roosts/total roosts in bioregion)	**100%** (22/22)	**100%** (10/10)	N/A (0/0)	N/A (0/0)	**100%** (184/184)	**100%** (1/1)

**Fig. 3:**
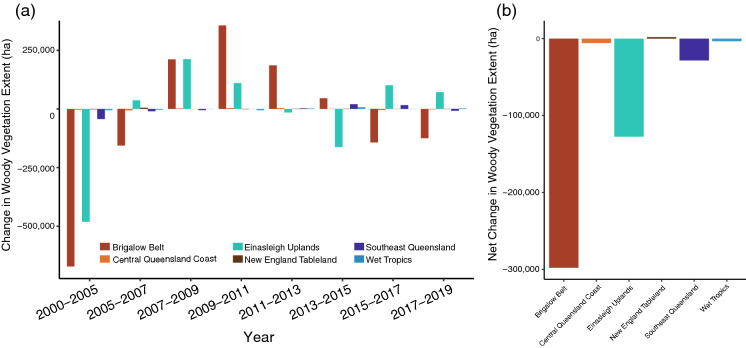
Change in Extent of Remnant Forests and Woody Vegetation in Bioregions of Interest 2000-2019. **a** Hectares lost or gained from the previous time point of woody vegetation in the bioregions important for black flying foxes. **b** Net change in woody vegetation from 2000 to 2019 by bioregion

Remnant woody vegetation showed a cycle of loss and growth over time, with an overall net loss of 460,866 hectares from 2000–2019 (Fig. 3). Remnant forest loss was greatest in the early 2000s. After 2007, major remnant vegetation regrowth was evident in the bioregions most affected by previous clearing: Brigalow Belt and Einasleigh Uplands (Fig. 3a). After 2013, both areas experienced reduced regrowth and some bioregions experienced a net loss of forest again. Coastal bioregions, such as Central Queensland Coast, Southeast Queensland, and the Wet Tropics show little change in the extent of remnant forests and woody vegetation over time (Fig. 3b).

**Fig. 4:**
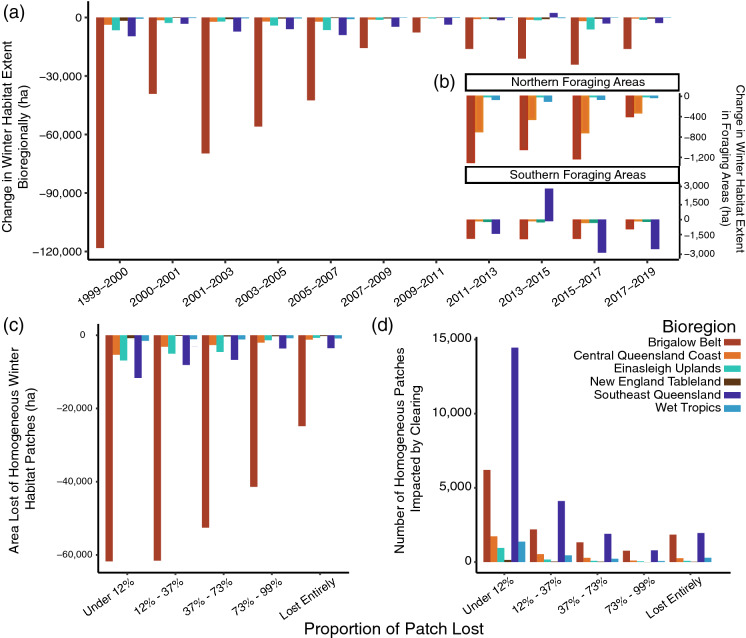
Extent Change of Winter Habitats for Black Flying Foxes and Homogenous Patch Loss Dynamics 1999-2019. Hectares lost or gained from the previous time point of VMRE maps colored by bioregion. **a** Extent change of possible winter habitats in bioregions of interest from 1999-2019. **b** Extent change of possible winter habitats for Northern and Southern foraging areas in 50 km around roosts from 2011-2019. **c** Extent loss of possible winter habitat homogenous patches binned by the proportion of the patch that was lost from 1999-2019. **d** Number of homogenous winter habitat patches that lost area, or were impacted by clearing, binned by the proportion of the patch that was lost from 1999-2019

We observed net losses of winter habitat for black flying foxes across all six of these bioregions (Fig. 4) and in roost foraging areas of 20 km, 50 km, and 80 km around roosts (Figure S1; Table S3). Here, we present results for changes in possible diet species and compare trends of habitat loss across bioregions and in the 50 km foraging areas around roosts (Fig. 4). A comparison of loss between major and possible diet species is presented in the SI. Winter habitat loss was greatest in Brigalow Belt, followed by Southeast Queensland with notably less in other bioregions. Habitat loss was greatest in the early 2000s but slowed down before reaching a low point in 2009 (Fig. 4a). However, clearing rates increased again after 2013. Collectively 537,038 ha of possible winter habitat was lost across all bioregions.

In 50 km foraging areas around winter roosts, there was a net loss of 6234 ha of possible winter habitat loss (0.65%) around Northern roosts and 9491 ha (0.38%) around Southern roosts from 2011 to 2019 (Fig. 4b). Prominently, southern foraging areas show a net increase in winter resources in Southeast Queensland between 2013 and 2015. However, this increase is an artifact of the mapping methodology because these patches were extant over the entire study period. After 2015, clearing rates increase again, and this bioregion became the greatest source of winter habitat loss in southern foraging areas.

We selected homogenous patches of winter habitat to characterize the area lost in patches dominated by winter resources for black flying foxes. We observed that clearing small extents of patches, typically less than 12–37% of their previously mapped size, caused the greatest losses of hectares (Fig. 4c) and impacted the greatest number of polygons (Fig. 4d). We also discerned that while more hectares of winter habitat were cleared in Brigalow Belt, a greater number of patches in Southeast Queensland were impacted by clearing and lost entirely from 1999 to 2019. These metrics together illustrate the high degree of forest fragmentation and loss that has already occurred in Southeast Queensland prior to our study, relative to other bioregions.

**Fig. 5:**
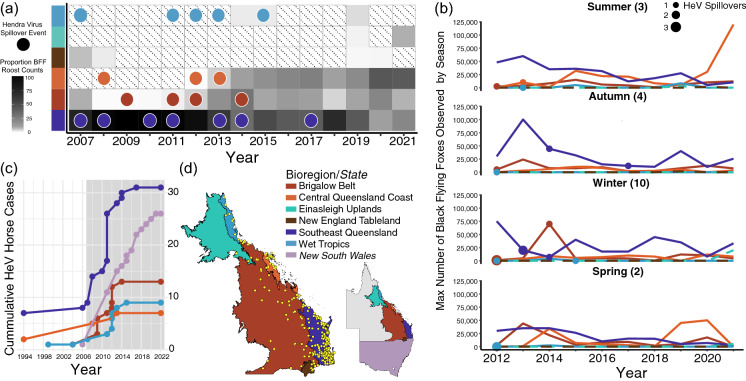
Observed Black Flying Fox Populations in the National Flying Fox Monitoring Program by Bioregion and Hendra Virus Spillovers. **a** Proportion of observed black flying fox roost counts seen in each bioregion from 2007 to 2021 with darker shades of grey representing a greater percentage of the total observed population, hatched cells representing no survey data, and circles denoting year and bioregion of HeV spillover events. **b** Number of black flying foxes observed in each bioregion by season since 2012. Filled circles represent the year and number of Hendra virus spillover events; size of circles corresponds to the number of confirmed horse cases. **c** Cumulative number of Hendra virus horse cases in each bioregion and state of New South Wales since detection with the grey box denoting the period shown in (**a**). **d** Bioregions of interest with roosts occupied by black flying foxes in winter as yellow dots; inset map of eastern Australia shows Queensland’s bioregions of interest and the state of New South Wales

Trends in black flying fox roost occupancy after 2007 revealed populations were mostly observed in Southeast Queensland, regardless of season (Fig. 5). In recent years though, growing percentages of the black flying population were observed in Brigalow Belt and Central Queensland Coast (Fig. 5a). Roost counts in New England Tableland, Wet Tropics, and Einasleigh Uplands were low and relatively stable after 2012. Although Southeast Queensland supported large roosts counts in most seasons and most years, the largest counts of black flying foxes in spring and summer have recently occurred in Central Queensland Coast (Fig. 5b). Hendra virus spillover to horses occurred most frequently during winter months (Fig. 5b) and in Southeast Queensland (Fig. 5c). Cases steadily increased in Brigalow Belt and the Wet Tropics before 2015. Since then, most spillovers have occurred just south of Queensland, in the state of New South Wales.

## Discussion

We mapped changes in remnant forests, winter habitats, human populations, and roost counts of black flying fox populations since 2000 in bioregions that support black flying foxes during winter. We found that human populations grew almost entirely near black flying fox roosts (Table 1; Fig. 2). Meanwhile, winter habitats and native forests showed net loss across the bioregions and particularly in roost foraging areas (Figs. 3, 4, S1). Winter habitat loss mostly occurred in Brigalow Belt (Fig. 4a), while most patches impacted by clearing, including size reduction and total loss, were in Southeast Queensland (Fig. 4d). Roost counts of observed black flying fox populations also declined at nationally monitored roosts in Southeast Queensland, while it increased in Brigalow Belt and Central Queensland Coast (Fig. 5a). Collectively, anthropogenic land pressure has led to native forest declines, winter habitat loss, and likely influenced black flying fox populations across these bioregions.

### Landscape changes

Our analyses showed human population growth, which was largely driven by migration (Australian Bureau of Statistics 2021), was overwhelmingly co-located with black flying fox foraging areas (Table 1; Fig. 2). These areas are known for their land productivity and have been sought out by humans for their fertile lands, mild climate and rainfall, and topographic characteristics (Lucas et al. 2008). Since these productive areas are preferred by both flying foxes and humans, we expected that they would experience the greatest loss of remnant forests and winter habitats. However, Brigalow Belt experienced the most remnant forest loss and winter habitat loss (Figs. 3, 4), despite comprising only 13% of the observed human population growth (Table 1). The majority of human population growth occurred in Southeast Queensland, but this bioregion only accounted for 6.l% of total winter habitat loss. We found that land pressure from an increasing human population did not directly cause the loss of remnant forests and native winter habitats. Rather, vegetation was mainly cleared for indirect human uses, including pasture and agriculture (Queensland Department of Environment and Science 2018). Regardless of foraging radius used, the percent of winter habitat loss from 1999 to 2019 was relatively consistent by bioregion (Figure S1; Table S3).

The rate of remnant forest loss and winter habitat loss was dependent on vegetation clearing policy in Queensland. In the Land Act of 1994, Queensland passed legislation to control clearing on leased and state-held land (Queensland Government 1994). In 2000, additional policies were enacted to control clearing on freehold land and leases (McGrath 2007). These new policies were met with resistance from landholders and sparked a phenomenon called ‘panic clearing’ by landholders, for fear of not being able to clear in the future (Simmons et al. 2018). We observed clearing that supports this theory, as winter habitat loss was highest in 1999 (Figs. 4, S2). Conversely, forest loss and winter habitat loss were lowest between 2007 and 2011, when a broadscale clearing ban was in effect. Clearing rates increased after this period when fewer permits were required for clearing (Queensland Government 2012). This shows that policy can be an effective tool to curb vegetation clearing and aid reforestation, but can also exacerbate clearing in the short term by influencing landholder behavior.

Fine-scale assessment of cleared winter habitats shows most patches lost a small percentage of their size, rather than being lost entirely (Fig. 5). This can increase the fragmentation of habitat or increase the edge-to-area ratio of patches, which increases edge effects (Broadbent et al. 2008; Bradshaw 2012) and soil degradation (Rasiah et al. 2004), and alters species’ competition dynamics (Grey et al. 1976). These factors may all negatively impact the quality of patches, potentially changing the productivity or timing of flowering. The effects of fragmentation on most winter diet species analyzed in this study are largely unknown. Moving forward, targeted studies are needed to document the impacts of fragmentation on flowering phenology and nectar quality in black flying fox winter diet species.

The decline in observed black flying fox roost counts after 2011 and increased use of roosts in Brigalow Belt and Central Queensland Coast (Fig. 5a) indicates the population is roosting in smaller numbers across a greater geographic area. Welbergen et al. 2020 tracked grey-headed, black, and little red flying foxes over five years and found that 60% of roosting sites were previously unknown and identified 123 potential new ‘colonies’ in Queensland and New South Wales. Eby et al. 2023 also described a decline in roost size with an increase in the number of roosts. Our results concur with these observations that black flying foxes are roosting in smaller populations and likely visiting new roosts, potentially not included in these surveys. Increased efforts to track and document black flying fox distribution and abundance across eastern Australia may help explain their declining numbers at regularly monitored sites.

### Implications for flying fox ecology and Hendra virus

While our measures of anthropogenic land pressure, forest loss, winter habitat loss, and declines in wildlife populations are correlative, evidence shows humans can influence flying fox foraging and roosting ecology; foraging studies of Australian flying foxes suggest the increased availability of resources in urban areas may attract grey-headed flying foxes (Roberts et al. 2012; Welbergen et al. 2020; Meade et al. 2021; Yabsley et al. 2021) and spectacled flying foxes (Tait et al. 2014) and entice them to roost in those areas continuously, rather than in less disturbed environments. In Bangladesh, roosting behaviors of *P. medius,* the main reservoir of Nipah virus in the ‘Nipah Belt’*,* showed populations existed in very small groups near human populations and exhibited largely sedentary behavior after centuries of land-use change (McKee et al. 2021). Nipah virus spillovers in Bangladesh, which occur from direct bat-to-human transmission, were found to be significantly associated with villages with higher human densities, greater forest fragmentation (Hahn et al. 2014), and winter months (McKee et al. 2021). Our findings here, consistent with Eby et al. 2023, suggest black flying fox populations may already be changing their roosting behavior and ecology similar to *P. medius* during ongoing habitat fragmentation and loss due to land-use change.

Removal of remnant forests and habitats has led to the persistent fissioning of flying fox populations (Eby et al. 2023), while also attracting them towards the increased reliability of urban resources near human populations (Meade et al. 2021; Yabsley et al. 2022). Increasing the availability of native forests and winter habitats as reliable resources could attract flying foxes away from urban environments (Plowright et al. 2021). Successful vegetation management and replanting strategies have been demonstrated at the Ku-ring-gai Flying Fox Reserve at Gordon, which has supported thousands of grey-headed flying foxes for decades (Pallin 2000; Ku-ring-gai Bat Conservation Society; 2020). Expanding these efforts across Queensland and New South Wales would potentially influence flying foxes to reduce urban residency time and begin returning to their historical nomadic behavior. This could geographically separate Hendra virus reservoir hosts from susceptible hosts in the human system and reduce nutritional stress on flying fox populations during the months when spillovers occur most frequently. Replanting winter diet species could also increase the likelihood of winter flowering pulses in the region, a crucial deterrent of Hendra virus spillover after consecutive years of food shortages (Eby et al. 2023).

Our analysis considered the maximum potential amount of remnant winter habitat extent and winter habitat loss, which almost certainly overestimates both. We included all heterogeneous patches with a winter diet species listed within the first five species of the vegetation community description. This may be too inclusive for some regional ecosystems, and resource abundance in some included regional ecosystems may be negligible for flying foxes. We only included mapped areas that meet the Queensland Herbarium’s definition of ‘remnant’ vegetation and spatial accuracy across the study area can vary. There were likely patches of habitat degraded below this standard that could have been productive winter habitats useful for flying foxes. We also acknowledge the sampling bias of the flying fox surveys toward previously established roosts in human-inhabited, accessible areas used by grey-headed flying foxes and spectacled flying foxes likely impacted the extent of our study area.

It will take a number of ecological and behavioral countermeasures to mitigate Hendra virus spillovers. Horse management, including keeping horses up to date on the Hendra virus vaccine and using overnight shelter to limit horse exposure to bat excreta, is necessary to reduce bat-horse spillover. Conservation of existing habitats and strategic replanting of winter diet resources will be essential for attracting flying foxes away from humans and horses and increasing foraging activity in native landscapes instead of agricultural lands (Giles et al. 2018; Eby et al. 2023). Conservation should be focused on patches containing species with relatively reliable annual productivity, or mature, contiguous canopies, which are likely to be more productive than smaller, fragmented areas (Law and Chidel 2008; Eby 2016). Although it is unknown how winter diet species will respond to future climate change scenarios, restoration efforts should consider the thermal and drought tolerances of species to ensure sufficient nectar productivity under future conditions. These efforts will also benefit other important pollinators, like honeyeaters and several bird species, other bat species, and small marsupials (Eby 2016). These ecological interventions will aid native wildlife, native eucalypt forests, and humans alike. Restoring native biodiversity and reducing the impact of human pressures on native environments will be essential for abating known and novel pathogen spillover to humans.

## Supplementary Information

Below is the link to the electronic supplementary material.

Supplementary file1 (DOCX 334 KB)

## Data Availability

The datasets generated during the current study are available in the HabitatLoss_BlackFlyingFox repository on the Bharti Lab Github here: https://github.com/bhartilab/HabitatLoss_BlackFlyingFoxes. The datasets analyzing during the current study are publicly available here: Regional Ecosystem Description Database: https://www.data.qld.gov.au/dataset/regional-ecosystem-description-database. Vegetation Management Regional Ecosystem 2019* Map: https://www.data.qld.gov.au/dataset/vegetation-management-act-series/resource/7fbb5561-7417-405f-8622-a4799687903b. *Previous maps of the Vegetation Management Regional Ecosystem Maps can be requested from the Queensland Herbarium. National Forest and Sparse Woody Vegetation: https://data.gov.au/dataset/ds-dga-69d09a6c-df77-439f-8bc7-87822cd520fd/details. WorldPop Human Population Count: https://hub.worldpop.org/geodata/listing?id=69; National Flying Fox Monitoring Program Queensland: https://www.data.qld.gov.au/dataset/flying-fox-monitoring-program.
