# Supplementary material for: Habitat loss for black flying foxes and implications for Hendra virus
Source: Landsc Ecol. 2023 Apr 5;38(6):1605–18. doi: 10.1007/s10980-023-01642-w (PMC10073794; doi:10.1007/s10980-023-01642-w)
Supplement: Supplementary file 1 — Supplementary file1 (DOCX 334 KB) [file 10980_2023_1642_MOESM1_ESM.docx]

**Habitat Loss for Black Flying Foxes and Implications for Hendra virus by Baranowski and Bharti**

**Supplementary Methods**

*Expansion of the Regional Ecosystem Description Database Table*

To identify vegetation communities that contained black flying fox winter diet resources, we used the Regional Ecosystem Description Database (REDD) Version 12 from the Queensland Herbarium (Queensland Herbarium 2019). Regional ecosystems are defined by a three-number identifier (e.g. 3.3.4) that corresponds to the bioregion, land zone, and vegetation community of a patch (Neldner et al. 2019). The Version 12 REDD table has 1,543 core regional ecosystems; however, some regional ecosystems have an additional letter further distinguishing vegetation communities within the same regional ecosystem (e.g. 3.2.4a).

We extracted all unique regional ecosystems written in the REDD table, resulting in 3,482 total regional ecosystems. We then acquired the list of all plant species in Queensland from the 2020 Census of Queensland flora list (Bean 2020). We further expanded this list to include all possible variations of species names including subspecies (e.g*. Eucalyptus melanophloia*, *E. melanophloia*, *Eucalyptus melanophloia* subsp. *melanophloia*, *E. melanophloia* subsp. *melanophloia*) to account for the variation in vegetation descriptions in the REDD table. We also included all genera and the phrase ‘sp’ and ‘spp’ (e.g. *Ficus* sp., *Ficus* spp.) in the list to account for uncertainty in species identification listed in the REDD table. This resulted in a list of 38,947 species or variations of floral species names.

Some regional ecosystems contain a descriptive phrase of the vegetation (e.g. “Simple to complex mesophyll to notophyll vine forest on moderately to poorly drained metamorphics (excluding amphibolites) of moderate fertility of the moist and wet lowlands, foothills and uplands.”) rather than any variation of floral species name. In such cases, we characterized the first sentence of the description as the first ‘species’ listed. We added these ‘species’ phrases into our unique list, totaling 39,066 variations of species names. We then extracted all corresponding species names and ‘species’ phrases on this list from the REDD table in order of appearance in the vegetation community description (see Table S1). This was essential as the order of species listed corresponds to the canopy layer in which they are found and the relative dominance of that species in the vegetation community (Neldner et al. 2019).

| RE_ID | Vegetation Description | Species1 | Species2 | Species3 | Species4 | Species5 | Species6 |
| --- | --- | --- | --- | --- | --- | --- | --- |
| 3.3.69 | Melaleuca dealbata and Corymbia clarksoniana (Clarkson's bloodwood) tall open forest. A sub-canopy includes C. clarksoniana, Livistona muelleri, and Acacia crassicarpa (spoon tree). Imperata cylindrica (blady grass) forms the dominant ground cover. Occurs on alluvial plains. (BVG1M-9e) | Melaleuca dealbata | Corymbia clarksoniana | C. clarksoniana | Livistona muelleri | Acacia crassicarpa | Imperata cylindrica |

Table S1. Example of Census of Queensland flora species extraction from Regional Ecosystem Description Database table.

We checked the accuracy of floral species names in the REDD table using Fuzzy Matching from the *tokenizers* pckg (Mullen et al. 2018). We counted the frequency of every word used in the vegetation descriptions, and then selected the words that were only used once to detect potentially misspelled words. We then found the string distance between words used only once and the unique species list using the *stringdist* pckg (van der Loo 2014). All floral species names from the REDD table that were one character apart from our species list were missing a space between the abbreviated genus and species name (e.g. *E.crebra* vs *E. crebra*). These misspelled species variations (n = 25) were added to the species list and we again extracted all floral species names in listed order from the REDD table.

*Black Flying Fox Winter Habitat Selection*

We identified regional ecosystems that contained at least one ‘major’ or ‘possible’ winter diet species, as observed from studies on black flying fox diet or diet of other flying fox species where there is species sympatry (Palmer 1999; Vardon et al. 2001; Markus and Hall 2004; Eby and Law 2008; Eby et al. 2019; Griffith 2020; Bell et al. 2021; Bradford et al. 2022). See Table S2 for list of major and possible black flying fox winter diet species. Since the abundance of productive vegetation within a patch is important for flying foxes (Eby and Law 2008), we only included regional ecosystems where the diet species of interest was listed within the top five species listed. This was to identify areas where these species were relatively ‘dominant’ or potentially of reasonable abundance to be useful for black flying fox populations.

*List of All Possible Black Flying Fox Winter Diet Species*

| **Species** | **Evidence** | **Area of Study** | **Study(s)** | **Major Diet Species** |
| --- | --- | --- | --- | --- |
| *Banksia integrifolia* | Observed feeding or feces | SEQ/NSW | Eby & Law 2008, Eby, Sims, & Bracks 2019, McWilliams 1985 | Yes |
| *Banksia* spp. | Feces eDNA metabarcoding | Northeast NSW | Griffith 2020 |  |
| *Callistemon* spp | Observed feeding | Brisbane/Urban | Markus & Hall 2004 |  |
| *Callistemon viminalis* | Feces eDNA metabarcoding | Charters Towers Roost QLD | Bradford personal comm., from Bell et al. 2021 & Bradford et al. 2022 | Yes |
| *Corymbia citriodora* | Observed feeding or feces | SEQ/NSW | Eby & Law 2008 | Yes |
| *Corymbia erythrophloia* | Feces eDNA metabarcoding | Charters Towers Roost QLD | Bradford personal comm., from Bell et al. 2021 & Bradford et al. 2022 | Yes |
| *Corymbia maculata* | Observed feeding or feces | SEQ/NSW | Eby & Law 2008, Eby, Sims, & Bracks 2019, McWilliams 1985 | Yes |
| *Corymbia polycarpa* | Feces eDNA metabarcoding | Charters Towers Roost QLD | Bradford personal comm., from Bell et al. 2021 & Bradford et al. 2022 | Yes |
| *Corymbia torrelliana* | Feces eDNA metabarcoding | Charters Towers Roost QLD | Bradford personal comm., from Bell et al. 2021& Bradford et al. 2022 | Yes |
| *Eriobotrya japonica* | Observed feeding | Brisbane/Urban | Markus & Hall 2004 | Yes |
| *Eucalyptus albens* | Observed feeding or feces | SEQ/NSW | Eby & Law 2008, Eby, Sims, & Bracks 2019 | Yes |
| *Eucalyptus camaldulensis* | Feces eDNA metabarcoding | Charters Towers Roost QLD | Bradford personal comm., from Bell et al. 2021 & Bradford et al. 2022 | Yes |
| *Eucalyptus conica* | Observed feeding or feces, not distinguished | New South Wales | Eby, Sims, & Bracks 2019 |  |
| *Eucalyptus crebra* | Feces eDNA metabarcoding | Charters Towers Roost QLD | Bradford personal comm., from Bell et al. 2021 & Bradford et al. 2022 | Yes |
| *Eucalyptus fibrosa* | Observed feeding or feces | SEQ/NSW | Eby & Law 2008 | Yes |
| *Eucalyptus fusiformis* | Observed feeding or feces, not distinguished | New South Wales | Eby, Sims, & Bracks 2019 |  |
| *Eucalyptus melliodora* | Observed feeding or feces | SEQ/NSW | Eby & Law 2008 | Yes |
| *﻿Eucalyptus miniata* | Observed feeding | Northern Territory | Palmer 1997, Vardon et al. 2001 |  |
| *Eucalyptus ochrophloia* | Observed feeding or feces, not distinguished | New South Wales | Eby, Sims, & Bracks 2019 |  |
| *Eucalyptus paniculata* | Observed feeding or feces, not distinguished | New South Wales | Eby & Law 2008, Eby, Sims, & Bracks 2019 |  |
| *Eucalyptus papuana* | Observed feeding | Northern Territory | Palmer 1997 |  |
| *Eucalyptus pilularis* | Observed feeding or feces | SEQ/NSW | Eby & Law 2008 | Yes |
| *Eucalyptus platyphylla* | Feces eDNA metabarcoding | Charters Towers Roost QLD | Bradford personal comm., from Bell et al. 2021 & Bradford et al. 2022 | Yes |
| *Eucalyptus polycarpa* | Observed feeding | Northern Territory | Palmer 1997 |  |
| *Eucalyptus robusta* | Observed feeding or feces | SEQ/NSW | Eby & Law 2008, Eby, Sims, & Bracks 2019 | Yes |
| *Eucalyptus seeana* | Observed feeding or feces | SEQ/NSW | Eby & Law 2008, Eby, Sims, & Bracks 2019 | Yes |
| *Eucalyptus siderophloia* | Observed feeding or feces | SEQ/NSW | Eby & Law 2008, Eby, Sims, & Bracks 2019 | Yes |
| *Eucalyptus sideroxlyon* | Observed feeding or feces | SEQ/NSW | Eby & Law 2008, Eby, Sims, & Bracks 2019 | Yes |
| *Eucalyptus* spp*.* | Feces eDNA metabarcoding | Northeast NSW | Griffith 2020 |  |
| *Eucalyptus tereticornis* | Observed feeding or feces | SEQ/NSW | Eby & Law 2008, Eby, Sims, & Bracks 2019 | Yes |
| *Eucalyptus tetradonta* | Observed feeding | Northern Territory | Palmer 1997, Vardon et al. 2001 |  |
| *Eucalyptus tetraplura* | Observed feeding or feces, not distinguished | New South Wales | Eby & Law 2008, Eby, Sims, & Bracks 2019 |  |
| *Eucalyptus xanthoclada* | Feces eDNA metabarcoding | Charters Towers Roost QLD | Bradford personal comm., from Bell et al. 2021 & Bradford et al. 2022 | Yes |
| *Ficus benjamina* | Observed feeding | Brisbane/Urban | Markus & Hall 2004 | Yes |
| *Ficus microcarpa* | Feces eDNA metabarcoding | Charters Towers Roost QLD | Bradford personal comm., from Bell et al. 2021 & Bradford et al. 2022 | Yes |
| *Ficus obliqua* | Observed feeding | Brisbane/Urban | Markus & Hall 2004 | Yes |
| *﻿Ficus racemosa* | feces below maternity roost | Northern Territory | Palmer 1997 |  |
| *Ficus* sp | Feces/rejecta, some from observation | Northeast NSW | McWilliams 1985, Griffith 2020, Bradford personal comm., from Bell et al. 2021 | Yes |
| *Ficus virens* | Observed feeding | Brisbane/Urban, NT | Markus & Hall 2004, Palmer 1997 | Yes |
| *Gravellia spp* | Observed feeding | Brisbane/Urban | Markus & Hall 2004 |  |
| *Lagunaria queenslandica* | Feces eDNA metabarcoding | Charters Towers Roost QLD | Bradford personal comm., from Bell et al. 2021 & Bradford et al. 2022 | Yes |
| *Ligustrum lucidum* | Feces eDNA metabarcoding | Northeast NSW | Griffith 2020 |  |
| *Macadamia* spp*.* | Feces eDNA metabarcoding | Northeast NSW | Griffith 2020 |  |
| *Mangifera indica* | Feces eDNA metabarcoding | Charters Towers Roost QLD | Bradford personal comm., from Bell et al. 2021& Bradford et al. 2022 | Yes |
| *Melaleuca fluviatilis* | Feces eDNA metabarcoding | Charters Towers Roost QLD | Bradford personal comm., from Bell et al. 2021& Bradford et al. 2022 | Yes |
| *Melaleuca leucadendra* | Feces eDNA metabarcoding | Charters Towers Roost QLD | Bradford personal comm., from Bell et al. 2021& Bradford et al. 2022 | Yes |
| *Melaleuca nervosa* | Feces eDNA metabarcoding | Charters Towers Roost QLD | Bradford personal comm., from Bell et al. 2021& Bradford et al. 2022 | Yes |
| *Melaleuca quinquenervia* | Observed feeding or feces | SEQ/NSW | Eby & Law 2008, Eby, Sims, & Bracks 2019, McWilliams 1985 | Yes |
| *Melaleuca* spp*.* | Feces eDNA metabarcoding | Northeast NSW | Griffith 2020 |  |
| *Melaleuca viridiflora* | Feces eDNA metabarcoding | Charters Town Roost QLD, Northern Territory | Bradford personal comm., from Bell et al. 2021, & Bradford et al. 2022, Palmer 1997 | Yes |
| *Melia azedarach* | Feces eDNA metabarcoding | Northeast NSW | Griffith 2020, Bradford personal comm., from Bell et al. 2021& Bradford et al. 2022 |  |
| *Nauclea orientalis* | Feces eDNA metabarcoding | Charters Town Roost QLD | Bradford personal comm., from Bell et al. 2021 & Bradford et al. 2022 | Yes |
| *﻿Nauclea orientalis* | feces below maternity roost | Northern Territory | Palmer 1997 |  |
| *﻿Passiflora foetida* | feces below maternity roost | Northern Territory | Palmer 1997 |  |
| *Solanum mauritianum* | Feces eDNA metabarcoding | Northeast NSW | Griffith 2020 |  |
| *Sygarus romanzoffiana* | Observed feeding | Brisbane/Urban | Markus & Hall 2004 | Yes |
| *Syncarpia glomulifera* | Observed feeding or feces, not distinguished | New South Wales | Eby & Law 2008, Eby, Sims, & Bracks 2019 |  |
| *﻿Timonius timon* | feces below maternity roost | Northern Territory | Palmer 1997 |  |
| *Ziziphus mauritiana* | Feces eDNA metabarcoding | Charters Towers Roost QLD | Bradford personal comm., from Bell et al. 2021& Bradford et al. 2022 | Yes |

Table S2. List of major and possible diet species for black flying foxes.

**Sensitivity Analysis of Foraging Radius**

We quantified winter habitat loss within a 20, 50, and 80 km radius of each roost known to support black flying foxes in winter (Figure S1). We find that the proportion of winter habitat loss was relatively equivalent in each bioregion, regardless of foraging radius used, with exception to Mulga Lands.

| **Bioregion** | **Brigalow Belt** | **Central Queensland Coast** | **New England Tableland** | **Southeast Queensland** | **Wet Tropics** | **Einasleigh Uplands** | **Mulga Lands** |
| --- | --- | --- | --- | --- | --- | --- | --- |
| Net Change in Winter Foraging Habitat Extent in 20km radius  Percent Change in Winter Foraging Habitat in 20km radius | -11,286.2  3.16% | -4008.6  -3.29% | -32.9  -0.76% | -28,235.1  -2.95% | -471.4  -1.65% | NA | NA |
| Net Change in Winter Foraging Habitat Extent in 50km radius  Percent Change in Winter Foraging Habitat in 50km radius | -56,248.3  -3.18% | -13,724  -3.12% | -1328.2  -2.06% | -45,832  -2.40% | -1150.1  -0.67% | -16.5  -0.03% | NA |
| Net Change in Winter Foraging Habitat Extent in 80km radius  Percent Change in Winter Foraging Habitat in 80km radius | -115,991.1  -3.22% | -15,909.3  -2.73% | -3820.9  -3.00% | -48,497  -2.33% | -2914.6  -1.02% | -2683.4  -0.69% | -3862.5  -35.8% |

Table S3. Change in Winter Habitat Extent within 20, 50, and 80 km foraging radii of roosts that supported black flying foxes in winter months between 2012 and 2020.

**
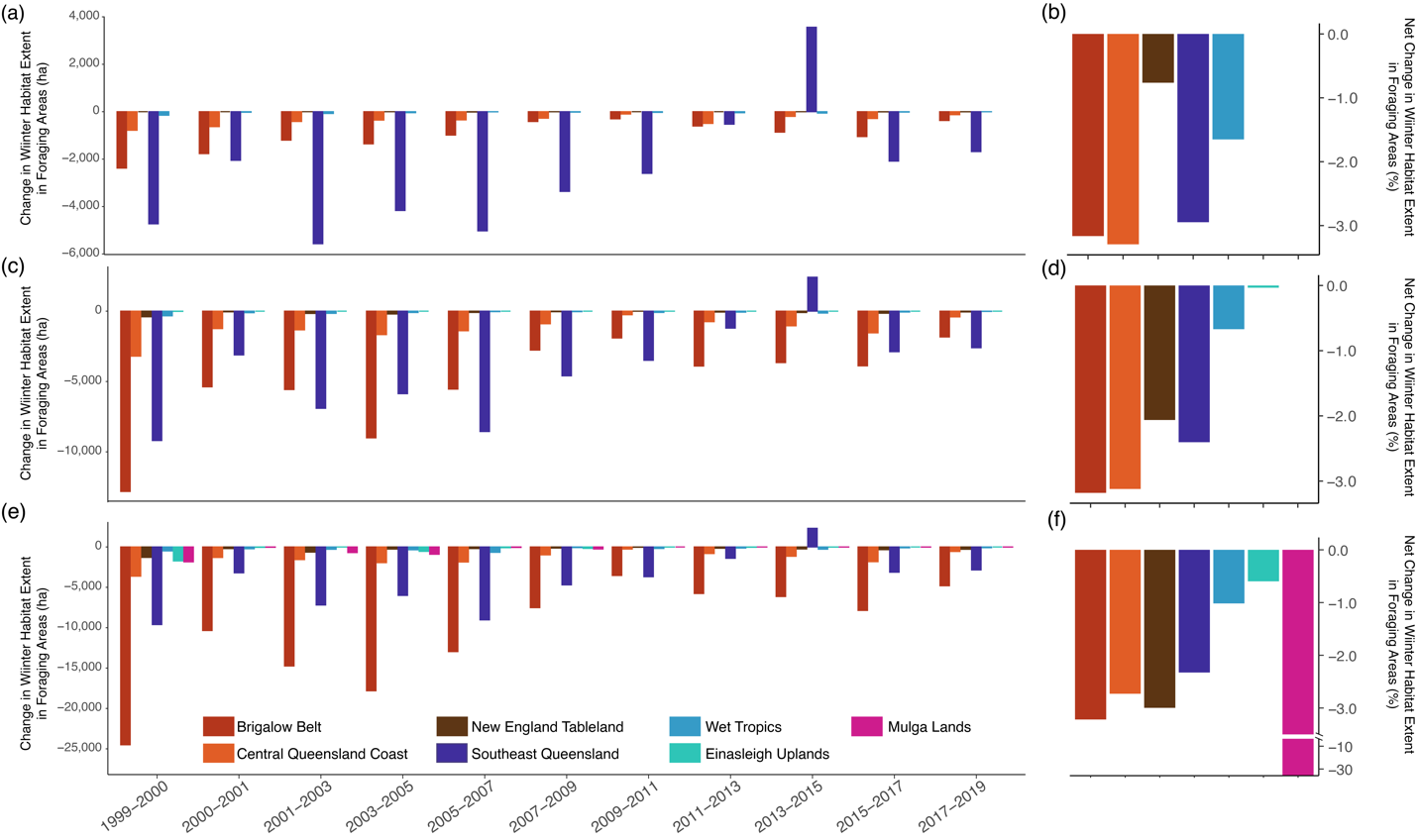
**

Figure S1. Extent Change of Winter Habitats for Black Flying Foxes in Foraging Areas 1999-2019. Hectares lost or gained from the previous time point of VMRE maps colored by bioregion. (a) Extent change of possible winter habitats in 20km foraging buffers around roosts 1999-2019. (b) Net change in winter habitat extent in 20km foraging areas1999-2019. (c) Extent change of possible winter habitats in 50km foraging buffers around roosts 1999-2019. (d) Net change in winter habitat extent in 50km foraging areas1999-2019. (e) Extent change of possible winter habitats in 80km foraging buffers around roosts 1999-2019. (f) Net change in winter habitat extent in 80km foraging areas1999-2019.

**Supplementary Results**

Differences in winter habitat loss between ‘major’ and ‘possible’ diet species were evident at the landscape scale (Figure S2a), but negligible at small spatial scales (Figure S2b). Northern foraging areas had no difference in habitat loss between diet lists. Southern foraging areas show a small net increase for major diet species from 2013 to 2015 however we acknowledge this is an artifact of the data. Similar to the bioregional trends of winter habitat loss, most patches lost less than 12% of their previously mapped size (Figure 4c, 4d).


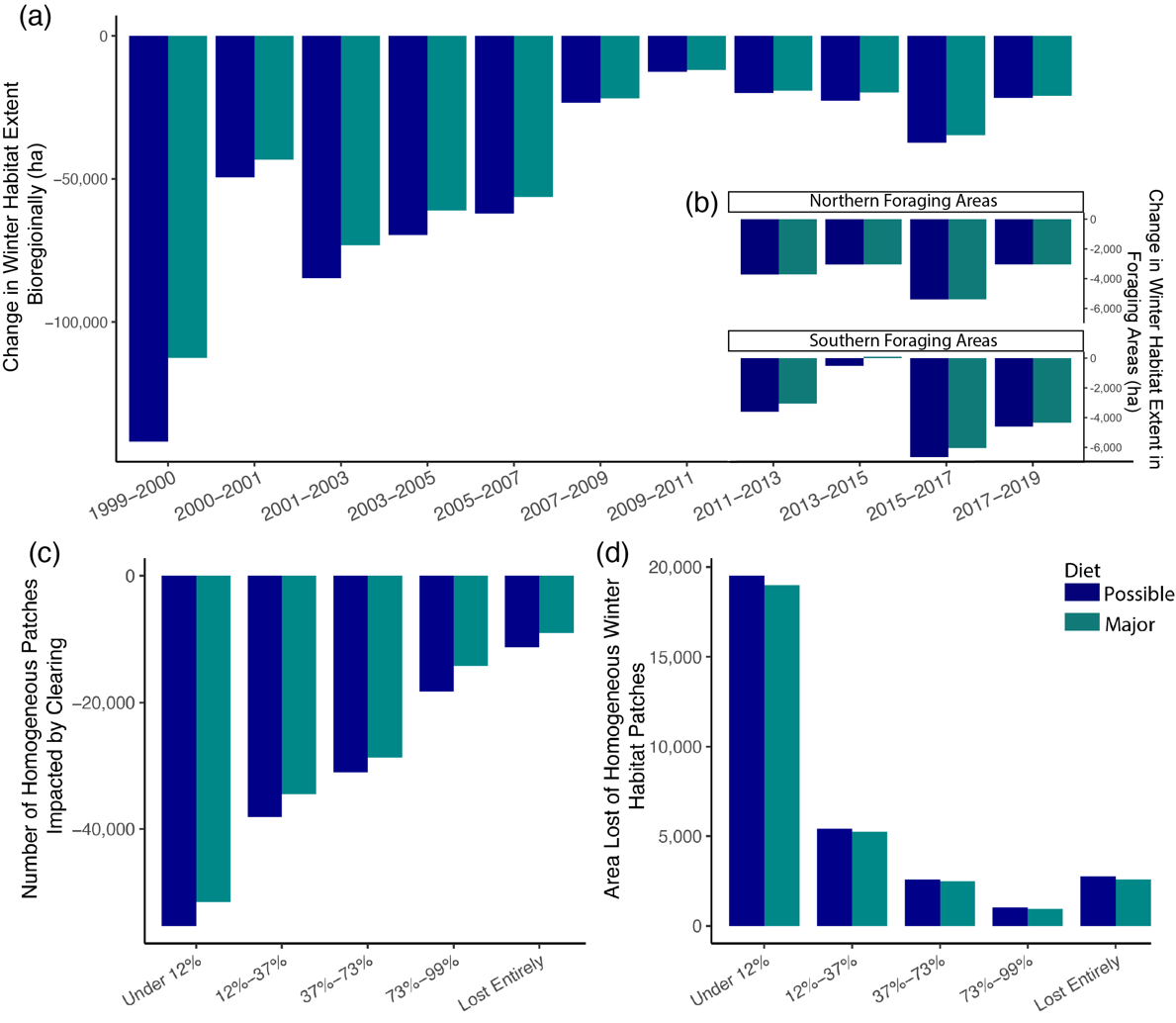


Figure S2. Extent Change of Winter Habitats for Black Flying Foxes and Homogenous Patch Loss Dynamics 1999-2019. Hectares lost or gained from the previous time point of VMRE maps colored by diet list. (a) Extent change of major (green) and possible (blue) winter habitats in bioregions of interest from 1999-2019. (b) Extent change of winter habitats for Northern and Southern foraging areas in 50km around roosts from 2011-2019. (c) Extent loss of winter habitat homogenous patches binned by the proportion of the patch that was lost. (d) Number of homogenous winter habitat patches that lost area, or were impacted by clearing, binned by the proportion of the patch that was lost.

**References**

A. Mullen L, Benoit K, Keyes O, et al (2018) Fast, Consistent Tokenization of Natural Language Text. J Open Source Softw 3:655. https://doi.org/10.21105/joss.00655

Bean AR (2020) Census of the Queensland Flora 2020. In: Dep. Environ. Sci.

Bell KL, Batchelor KL, Bradford M, et al (2021) Optimisation of a pollen DNA metabarcoding method for diet analysis of flying-foxes (Pteropus spp.). Aust J Zool

Bradford M, Venz M, Bell KL, et al (2022) The diet of a specialist nectarivore in Australia: The little red flying-fox (Pteropus scapulatus, Pteropodidae). Austral Ecol 47:619–628. https://doi.org/10.1111/aec.13143

Eby P, Law B (2008) Ranking the feeding habitats of Grey-headed flying foxes for conservation management. A report for The Department of Environment and Climate Change (NSW) & The Department of Environment, Water, Heritage and the Arts

Eby P, Sims R, Bracks J (2019) Flying-fox Foraging Habitat Mapping NSW spatial patterns of habitat quality for flying-foxes

Griffith P (2020) Diet Partitioning in newly sympatric urban flying-foxes (Pteropus poliocephalus and Pteropus alecto). Aust Mammal 42:361–366

Markus N, Hall L (2004) Foraging behaviour of the black flying-fox (Pteropus alecto) in the urban landscape of Brisbane, Queensland. Wildl Res 31:345–355. https://doi.org/10.1071/WR01117

Neldner VJ, Wilson BA, Dillewaard HA, et al (2019) Methodology for survey and mapping of regional ecosystems and vegetation communities in Queensland.Version 5.0. Updated March 2019. Queensl Herb Queensl Dep Environ Sci Brisbane

Palmer C (1997) Ecology of the black flying-fox Pteropus alecto in the seasonal tropics of Northern Territory: Resource tracking in a landscape mosaic and role in seed dispersal. MS Thesis School of Biological and Environmental Sciences, Northern Territory University, Australia.

Queensland Herbarium (2019) Regional Ecosystem Description Database (REDD). Version 12.1

van der Loo M (2014) The stringdist package for approximate string matching. R J 6:111–122

Vardon MJ, Brocklehurst PS, Woinarski JCZ, et al (2001) Seasonal habitat use by flying-foxes, Pteropus alecto and P. scapulatus (Megachiroptera), in monsoonal Australia. J Zool 253:523–535. https://doi.org/10.1017/S0952836901000486
